# Reduction of Serum ADAM17 Level Accompanied with Decreased Cytokines after Abatacept Therapy in Patients with Rheumatoid Arthritis

**Published:** 2014-12

**Authors:** Masayu Umemura, Takeo Isozaki, Syo Ishii, Shinya Seki, Nao Oguro, Yoko Miura, Yusuke Miwa, Masanori Nakamura, Katsunori Inagaki, Tsuyoshi Kasama

**Affiliations:** 1 Division of Rheumatology, Department of Medicine, Showa University School of Medicine, Tokyo, Japan;; 2 Department of Orthopedics, Showa University Northern Yokohama Hospital, Yokohama, Japan;; 3 Department of Orthopedics, Showa University School of Medicine, Tokyo, Japan

**Keywords:** Rheumatoid arthritis, a disintegrin and metalloprotease 17, cytokine, abatacept

## Abstract

A disintegrin and metalloprotease 17 (ADAM17) appears to be recognized as an important player in tissue destruction and also exacerbation of inflammation related with increased activities of angiogenesis in several pathological conditions. To examine the modulation of serum levels of ADAM17 and inflammatory cytokines in patients with rheumatoid arthritis (RA) in response to therapy of abatacept (ABT). Twenty four patients with RA were enrolled in our study. Serum was collected immediately prior to (baseline) and 24 weeks after starting ABT therapy. Serum levels of ADAM17 and cytokines/chemokine were quantified using enzyme-linked immunosorbent assay. ADAM17 level was markedly higher in RA patients than in healthy individuals. Positive correlation was observed between the baseline ADAM17 and CX3CL1 at baseline. There was a significant overall reduction of RA disease activity (Disease Activity Score 28) from 4.73 to 2.79 after 24 weeks after the ABT therapy. Furthermore, there was a significant reduction in serum level of ADAM17 in RA patients, and the patients achieved clinical responses, and also clinical remission had a significant decrease in ADAM17 level and also levels of tumor necrosis factor α, IL-6 and CX3CL1 after 24 weeks of ABT therapy. Our results suggest that the suppression of ADAM17 secretion and function seems to be a crucial therapeutic target in the treatment of ABT in patients with RA.

## INTRODUCTION

The complicated networks between inflammatory/immune cells and pro-inflammatory molecules with potentially important roles have been implicated in the pathogenesis of rheumatoid arthritis (RA) (1-3) . Numerous mediators, including inflammatory cytokines and adhesion molecules, have been implicated in this process (2, 4) . In a recent decade, the crucial therapeutic targets to inhibit the disease activities of RA were focused to these cytokines such as tumor necrosis factor (TNF) and IL-6 (5-7) . In addition, it has been more recently demonstrated that the inhibition of T lymphocyte activation was recognized as an effective to suppress RA activities. Abatacept (ABT) is a fusion protein that consists of the extracellular domain of human cytotoxic T-lymphocyte-associated antigen 4 (CTLA-4) linked to a modified Fc portion of human immunoglobulin G1 (IgG1), and selectively modulates the CD80/CD86:CD28 costimulatory signal, resulting the inhibition of activation and proliferation of T lymphocytes (8). ABT has introduced clinical improvement in signs and symptoms, disability, and significantly inhibited the progression of joint damage in early and long-standing disease (9).

A disintegrin and metalloprotease 17 (ADAM17) or tumor necrosis factor-α converting enzyme (TACE) was first described as the protease responsible for TNFα shedding (10, 11), and this enzyme was involved in the physiological cleavage of membrane-anchored cytokines, including TNFα and fractalkine (CX3CL1) (10-12), releasing it in soluble form. ADAM17 was also shown to solubilize a wide variety of proteins including TNF receptors (R) (13) , and IL-6R (14). In addition, the implication of ADAM17 substrates in immunoregulation has made this enzyme an efficient therapeutic target in the treatment of a number of pathological conditions including airway inflammation, cancer, and arthritis (15, 16) . Also, an elevated level of ADAM17 expression was found in synovial tissue and mainly in macrophage- and fibroblast-like synovial cells and also cartilage in patients with RA, as compared with patients with osteoarthritis, and with normal cartilage (17-19) , suggesting that abnormal ADAM17 activity and expression may contribute to the development of several pathological conditions, including RA.

The aim of the present study was to examine the modulation of ADAM17 and also inflammatory cytokines/chemokines including TNFα and CX3CL1 in patients with RA in response to therapy of ABT, since ADAM17 is the predominant protease catalyzing the release of crucial cytokines that are involved in a cascade of events leading to RA.

## MATERIALS AND METHODS

### Patients

The study design was a prospective observational cohort study. Twenty four patients with RA and who fulfilled the 1987 American College of Rheumotology (ACR) criteria (20) were enrolled in our study between 2011 and 2013. All patients failed to respond to treatment with methotrexate (MTX) or other either biologic- or non-biologic disease modifying anti-rheumatic drugs (DMARDs), and patients who were needed to consider the changes of treatments by judged by the treating physician were also enrolled. Glucocorticoids (<10 mg/day of prednisolone) and non-steroidal anti-inflammatory drugs had to have been given at a stable dose for at least 4 weeks (wks) prior to enrollment and during the course of treatment in the study. Patients received intravenous ABT at a dose of 500 mg or 750 mg (≤60 kg or >60 kg of body weight, respectively) at baseline and after 2, 4 and every 4 weeks. Disease activity and its clinical improvement were assessed using Disease Activity Score (DAS28) [erythrocyte sedimentation rate (ESR) 4] with European League Against Rheumatism (EULAR) response criteria (21). Remission was defined as DAS28-ESR < 2.6 after 24 wks of treatment. Functional ability was assessed with the modified Health Assessment Questionnaire (mHAQ) score. In the period of study (24 wks from therapy), there was no alteration of their doses of medications including MTX, other DMARDs and prednisolone.

### Serum samples

Serum was collected immediately prior to (baseline) and 24 wks after starting ABT therapy. In addition, control serum was obtained from twenty four age- and sex-matched healthy volunteers (n=24). Serum rheumatoid factor (RF), matrix metalloproteinase (MMP-3), C-reactive protein (CRP) levels, and ESR were determined using a latex photometric immunoassay and the Westergren method, respectively. Anti-cyclic citrullinated protein antibody (ACPA) was measured using commercial double ligand enzyme-linked immunosorbent assays (ELISA) kit (Diastat Anti-CCP; MBL Tokyo, Japan).

All experiments were carried out in accordance with protocols approved by the Human Subjects Research Committee at our institution, and all human experiments were performed in accordance with the Declaration of Helsinki, and informed consent was obtained from all patients and volunteers.

### Determination of serum levels of ADAM17 and cytokines

Serum levels of ADAM17 and the cytokines, including TNFα, IL-6, CX3CL1 (fractalkine), CXCL8 (IL-8), CCL2 (monocyte chemoattractant protein-1), CCL3 (macrophage inflammatory protein-1 alpha) were quantified using commercial ELISA kits according to the manufactures’ instructions (R&D Systems, Minneapolis, MN, USA).

Because it is possible that the anti-IgG activity of the RF in the samples may have augmented the reactivity of the ELISA systems for cytokines and ADAM17, we also assayed ADAM17 and cytokines in five randomly chosen serum samples before and after depleting RF using RF Stripper (Binding Site, Birmingham, UK). No significant differences in serum levels of all cytokines were found between the RF-depleted and untreated sera (data not shown).

### Statistical analysis

Data were expressed as means ± standard deviation of the mean. The differences between groups were evaluated using the Mann-Whitney U-test. Follow-up data were evaluated using Wilcoxons signed rank test. The relationship between serum cytokine levels and the RA disease activity and indicated measures was evaluated using the Speaman rank correlation. Values of *p*<0.05 were considered significant.

## RESULTS

### Serum ADAM17 levels in RA patients

**Figure 1:**
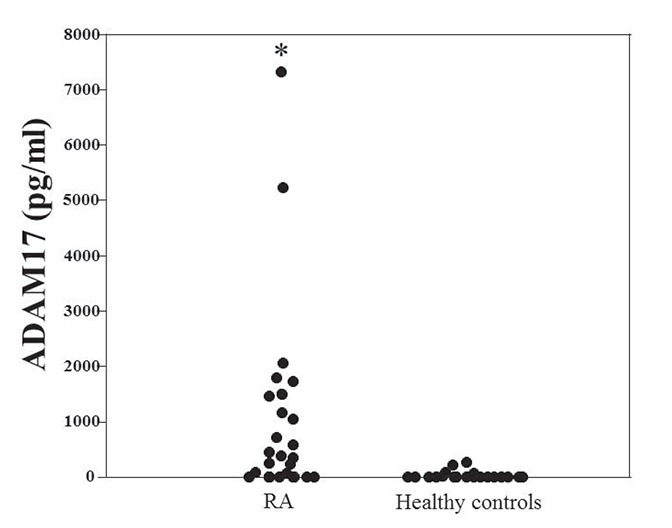
Serum ADAM17 level in patients with RA and healthy individuals. Serum was obtained from patients at baseline and healthy individuals. Serum ADAM17 was assayed by ELISA. *p<0.0001 vs healthy individuals (controls).

**Figure 2:**
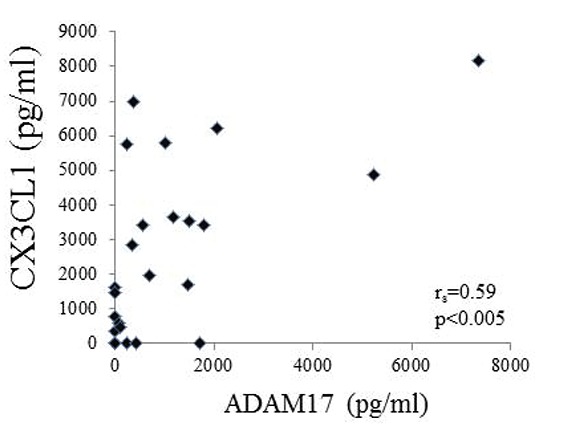
Positive correlation between serum levels of ADAM17 and CX3CL1. Serum was obtained from patients at baseline. Serum ADAM17 was assayed by ELISA. Each point represents a sample collected from a different RA patient. Positive correlation was observed between the baseline ADAM17 and CX3CL1 (Spearman's rank correlation coefficient [*r_s_*] = 0.59, *p*<0.005).

The patient characteristics are summarized in Table 1. Twenty-four RA patients with ABT therapy were enrolled in the present study. At the start of therapy, the mean age of the patients was 64.7 years, disease duration was 10.4 years, and the baseline DAS28 was 4.73. We initially used ELISA to compare the ADAM17 levels in serum samples from all RA patients and from healthy individuals (n=24). ADAM17 levels were markedly higher in RA patients than in healthy controls (Fig.1; *p*<0.0001). At baseline, there was no significant correlation between the serum levels of ADAM17 and either RA disease activity or serum measures including RF, ACPA, and MMP-3 (data not shown). Because it is known that ADAM17 is involved in the physiological cleavage of membrane-anchored inflammatory cytokines, serum cytokine levels were examined in patients with RA at baseline. Positive correlation was observed between the baseline ADAM17 and CX3CL1 (CX3CL1: Spearman’s rank correlation coefficient [*r_s_*] = 0.59, *p*<0.005, Fig. 2). In addition, the weak correlation was observed between ADAM17 and TNFα (*r_s_*=0.41, *p*=0.05), although there was no significant correlation between the baseline ADAM17 and IL-6.

**Table 1:** Patient characteristics and clinical responses by group

	All	Responsive group	Unresponsive group	*p*	Remission	Non-remission	*p*

Patients (male/female)	24 (5/19)	21	3		10	14	
Age (yrs)	64.7 ± 10.9	65.9 ± 9.0	56.0 ± 20.3	n.s.	61.0 ± 13.2	67.3 ± 8.3	n.s.
Disease duration (yrs)	10.4 ± 1.9	6.4 ± 5.0	11.6 ± 17.6	n.s.	6.0 ± 5.1	7.8 ± 8.5	n.s.
Dosage of MTX (mg/wk)	8.3 ± 5.0	8.3 ± 4.9	8.0 ± 6.9	n.s.	10.1 ± 4.1	7.0 ± 5.4	n.s.
% positive	83.3	85.7	66.7	n.s.	100.0	71.4	n.s.
Prednisolone (mg/day)	2.9 ± 2.7	3.0 ± 2.7	2.0 ± 2.6	n.s.	2.6 ± 2.0	3.0 ± 3.2	n.s.
% positive	66.7	71.4	66.7	n.s.	70.0	64.3	n.s.
ESR (mm/h)	30.3 ± 19.3	31.2 ± 19.7	24.0 ± 19.1	n.s.	24.3 ± 18.2	34.6 ± 19.6	n.s.
CRP (mg/dl)	1.7 ± 2.2	1.7 ± 2.2	1.4 ± 2.2	n.s.	1.8 ± 2.7	1.5 ± 1.8	n.s.
TJC	10.9 ± 1.8	8.0 ± 4.6	3.3 ± 4.0	n.s.	4.9 ± 3.5	9.1 ± 4.8	n.s.
SJC	6.2 ± 0.8	5.3 ± 4.0	3.0 ± 4.4	n.s.	4.1 ± 5.0	5.7 ± 3.2	n.s.
DAS28 (ESR-4)	4.7 ± 1.5	5.0 ± 1.3	3.1 ± 2.1	n.s.	3.8 ± 1.7	5.4 ± 0.8	n.s.
mHAQ	1.5 ± 1.5	1.7 ± 1.6	1.0 ± 1.7	n.s.	0.2 ± 0.3	2.5 ± 1.3	<0.0001
RF (IU/ml)	114.9 ± 168.8	104.6 ± 157.4	186.6 ± 266.6	n.s.	122.4 ± 223.8	109.5 ± 125.3	n.s.
% positive	87.5	90.5	66.7	n.s.	70.0	100.0	n.s.
MMP-3 (ng/ml)	244.7 ± 212.4	263.9 ± 219.2	110.3 ± 85.4	n.s.	205.7 ± 260.2	272.5 ± 175.9	n.s.
ACPA (U/ml)	343.9 ± 472.6	387.5 ± 490.5	38.5 ± 50.3	n.s.	45.2 ± 63.5	557.3 ± 524.2	<0.005
% positive	79.2	76.2	66.7	n.s.	60.0	85.7	n.s.
Biologics-naïve pts (n)	8	8	0		4	4	

ACPA, anti-cyclic citrullinated protein antibody; CRP, C-reactive protein; DAS28, disease activity score 28; ESR, erythrocyte sedimentation rate; mHAQ, modified health assessment questionnaire; MMP-3, matrix metalloproteinase-3; MTX, methotrexate; n.s., not significant; pts, patients; RF, rheumatoid factor; SJC, swollen joint count; TJC, tender joint count; wk, week; yrs, years.

### Reduction of ADAM17 levels with response to ABT therapy in patients with RA

In RA patients, there was a significant overall reduction of RA disease activity (DAS28) from 4.73 ± 1.48 to 2.79 ± 1.07 (*p*<0.0001) after 24 wks after the ABT therapy. In addition, 21 patients treated with ABT achieved moderate and good responses, and remission (DAS28-ESR<2.6) was induced in 10 patients. However, there were no significant differences in patient characteristics and laboratory parameters at baseline between the remission group and nonremission group, except for mHAQ sores and ACPA titers, while no significant differences were seen in all parameters between the responsive and unresponsive groups (Table 1).

**Figure 3:**
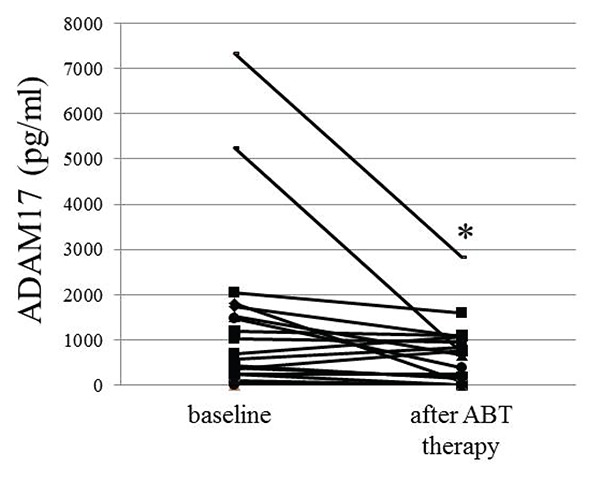
Effect of ABT therapy on ADAM17 level in RA patients Serum was obtained from patients at baseline and after 24 weeks of ABT therapy. A significant reduction in serum ADAM17 level was observed after ABT treatment (*p<0.05).

**Figure 4:**
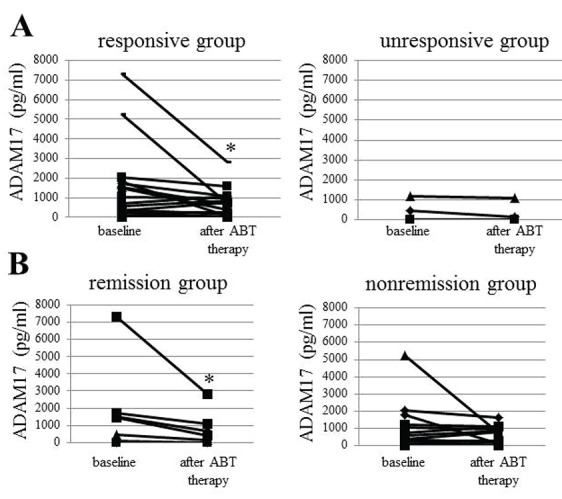
The relation between changes in serum ADAM17 level and clinical responses to ABT therapy. Panel A: A significant reduction in serum ADAM17 level was observed in the responsive group (n=21, *p<0.05), but not in the unresponsive group. Panel B: Serum ADAM17 levels were significantly decreased in the group achieved clinical remission (n=10, *p<0.05) after 24 weeks of ABT therapy.

We next examined the changes of ADAM17 levels in patients with ABT therapy. After ABT therapy, there was a significant reduction (*p*<0.05) in serum levels of ADAM17 in RA patients, although ADAM17 level in 5 patients was below a detection level (<10 pg/ml as zero value) of ELISA at both baseline and after medication (Fig. 3). We next analyzed the relation between changes in serum ADAM17 levels and clinical responses to ABT therapy. There was a significant reduction in serum ADAM17 levels in the responsive group (n=21, 1182.7 ± 1858.9 (baseline) to 543.8 ± 710.0 pg/ml, *p*<0.05), but not in the unresponsive group (Fig. 4A). Furthermore, serum ADAM17 levels were significantly decreased in the group (n=10, 1257.1 ± 2254.6 vs. 509.1 ± 892.5 pg/ml, *p*<0.05) achieved clinical remission after 24 wks of ABT therapy (similarly ADAM17 level in 5 patients was below a detection level at both baseline and after medication) (Fig. 4B), although there were no statistically significances for baseline ADAM17 levels between any response groups (data not shown). Because patient groups are heterogeneous such as the primary and secondary non-responder groups to prior biologics (TNF or IL-6 antagonists) and biologics-naïve groups, we examined the alteration of DAM17 in only biologic-naïve patients (n=8). ADAM17 was decreased from 1352.6 ± 1756.8 pg/ml to 442.1 ± 551.4 pg/ml (*p*<0.05) in biologic-naïve patients after ABT therapy, although there was no significant reduction of ADAM17 in the unresponsive group with prior biologics.

At last, changes of serum cytokine levels were examined after 24 wks of ABT therapy. As well as ADAM17, serum concentrations of TNFα, IL-6 and CX3CL1 were significantly decreased in all RA patients and also patients with clinical responses after 24 wks of ABT therapy, as shown in Table 2. While, there were no significant changes of serum concentrations of other chemokines, including CXCL8, CCL2, and CCL3 after ABT therapy in either all RA patients or those with any clinical responses (data not shown).

**Table 2:** Changes in serum concentrations of ADAM17 and cytokines in RA patients taking ABT therapy

	All RA patients (n=24)			Responsive group (n=21)			Unresponsive group (n=3)		
	Baseline	24 wks	*p*	Baseline	24 wks	*p*	Baseline	24 wks	*p*

TNFα (pg/ml)	87.9 ± 203.5	17.1 ± 25.4	<0.01	99.6 ± 215.6	18.6 ± 26.9	<0.005	6.2 ± 5.7	7.0 ± 6.1	n.s
IL-6 (pg/ml)	23.5 ± 37.5	8.7 ± 22.8	<0.05	26.4 ± 39.3	9.5 ± 24.3	<0.01	3.4 ± 2.0	3.5 ± 4.9	n.s
CX3CL1 (pg/ml)	2648.6 ± 2518.9	1986.0 ± 2868.8	<0.05	2836.1 ± 2569.5	2053.4 ± 3032.0	<0.05	1336.5 ± 1996.8	1514.4 ± 1526.7	n.s

ABT, abatacept; ADAM17, a disintegrin and metalloprotease domain 17; CX3CL1, chemokine (C-X3-C motif) ligand 1; IL-6, interleukin-6; TNFα, tumor necrosis factor α; n.s., not significant.

## DISCUSSION

It has been known that enzymes such as ADAM as well as MMP, play a crucial role in the destruction of joint structures including bone and cartilage in RA synovitis (22). ADAM10 is one of an important enzyme, and is observed in synovial tissues and fluids in patients with RA (23, 24) . Recently, ADAM17 appears to be recognized as another important player in tissue destruction and also exacerbation of inflammation related with increased activities of angiogenesis, as well as ADAM10. ADAM17 constitutively expressed by several cell types, including T cells, monocytes, endothelial cells, and fibroblasts (10) , and increased expression of ADAM17 was observed in synovial tissue, as compared with patients with osteoarthritis, and was mainly seen in macrophage- and fibroblast-like synovial cells (18, 19) . Furthermore, it has been demonstrated that ADAM17 is induced upon exposure to hypoxia and TNFα, which are important factors in rheumatoid synovitis (17, 19). In the present study, we showed that serum concentration of ADAM17 was increased in patients with RA in compared to normal individuals, and a significant correlation between ADAM17 and CX3CL1, which is involved in the pathogenesis of rheumatoid synovitis and also related vasculitis (25-27) , was seen in RA patients at baseline. It is well known that ADAM17 is a crucial protease responsible for the conversion of TNFα and CX3CL1 from a membrane-bound precursor to a soluble cytokine (10-12, 28) . In the present study, it has been shown that there was a significant reduction in serum ADAM17 levels in the responsive group, but not in the unresponsive group, and also in patients with a clinical remission after ABT therapy. In addition with ADAM17, similar decreases of serum levels of TNFα, IL-6 and CX3CL1 were observed after ABT therapy in RA patients (Table 2). ABT induces the decrease in peripheral blood of cytokine (IL-17 and IFN-γ)-producing T cells and in serum IFN-γ in response with improved disease activity (29-31). In this regard, Marti et al. have reported that no significant reduction of serum TNFα, levels was seen in patients after ABT (CTLA4Ig) therapy (30), in contrast with our results. These conflict data in cytokine modulation in serum may be due to differences in the short observational periods (12 wks) and small sample numbers (n=7) in the report by Marti, et al. Collectively, ADAM17 would be an important to modulate cytokine expression and to cleavage from cells, and also seems to be sensitive for effectiveness of ABT therapy in patients with RA.

On the other hand, ADAM17 mRNA has been shown to be highly expressed in the thymus and spleen, and high ADAM17 protein levels have been detected in T cells, as described above, suggesting that ADAM17 may play an important role in T cell development and/or function (10, 32) . Indeed, ADAM17 expression was augmented by T cells in response to bacterial (*Porphyromonas gingivalis*) stimulation (33), indicating that T cell activation is crucial factor for stimulated expression of ADAM17. Recently, it has been demonstrated that circulating CD4+CD28- T cells decrease in parallel with decreased RA activity in response with ABT (31, 34, 35). In the present study, serum concentration of ADAM17 was reduced after ABT therapy in RA patients, suggesting that deactivation and decreased numbers of T lymphocytes induced by ABT may be in part responsible for the reduction of ADAM17 levels.

Taken together, the orchestration and modulation of inflammatory cytokines via ADAM17 may account for the augmentation of abnormal inflammatory/immune responses in the pathogenesis of RA synovitis. Moreover, ABT may be induced to improve synovial inflammation through terminating inflammatory cytokine networks by, in part, inhibiting ADAM17 and consequently disease activity in RA patients as well as the inhibition of T lymphocyte activation, although direct mechanisms or interaction of inflammatory mediators such as TNFα, IL-6 and CX3CL1 by ABT therapy have not been still resolved in the present study. Since the limitations of the present study are that the number of enrolled patients is relatively small, future studies should be performed in the large number of RA patients to confirm the pathophysiological role of ADAM17 in the course of RA with biologics such as ABT or antagonists for TNF or IL-6. Finally, the inhibition of ADAM17 expression and secretion seems to be a crucial therapeutic target in the treatment of ABT in patients with RA.
